# Supplementary material for: Evaluating indoor residual spraying in Côte d’Ivoire: the role of routine malaria data quality
Source: Int J Public Health. 2026 Oct 1;71:1608766. doi: 10.3389/ijph.2026.1608766 (PMC13669831; doi:10.3389/ijph.2026.1608766)
Supplement: Supplementary file 1 [file Supplementaryfile1.docx]

**Supplementary Material**

Supplementary Figure 1: Missing data of contemporaneous covariates in the four districts of the study.

| **Variable name** | **Description** | **Source** | **monthly** | **yearly** |
| --- | --- | --- | --- | --- |
| Rainfall | Total precipitation in m | ERA5 | X |  |
| Temperature | 2m temperature in degree Celsius | ERA5 | X |  |
| Leaf | Leaf area index, low vegetation | ERA5 | X |  |
| Humidity | 2m dewpoint temperature in degree Celsius | ERA5 | X |  |
| biting_rate_hcl_indoor | Indoor biting rate of An. gambiae and funestus combined | PMI-Vectorlink | X |  |
| biting_rate_hcl_outdoor | Outdoor biting rate of An. gambiae and funestus combined | PMI-Vectorlink | X |  |
| EIR_bpn | Infectious bites per night per person | PMI-Vectorlink | X |  |
| N_hf_active | Number of health facilities reporting cases during a month per 1000 population | DHIS2 | X |  |
| hf_reporting_rate | Number of consecutive months reporting cases per months from first report until end of year | DHIS2 |  | X |
| Testing | Number of tested (RDT or blood spot) per suspected cases | DHIS2 | X |  |
| tpr_total | Number of positive tested per total number of tested (RDT or blood spot) | DHIS2 | X |  |
| ipt1_coverage | Number of IPT doses administered during first trimester ANC visit per total number of first trimester ANC visits | DHIS2 |  | X |

Supplementary Table 1: Description of covariates used in the causal impact analysis by source and periodicity.

| Adjustment | Description | Details | Formula | Data sources |
| --- | --- | --- | --- | --- |
| 0 | crude incidence | uncomplicated & complicated cases | N0 | DHIS2, PMI |
| 1 | presumed (**pr**) & test postivity rate (**tpr**) | Add cases that have not been confirmed (i.e. presumed) by assuming that their chance to test positive is as **tpr** | N1=N0+pr*tpr | DHIS2 |
| 2 | reporting rate (**rr**) | Take into account the annual reporting rate | N2=N1/rr | DHIS2 |
| 3 | health care seeking (**s**) | Add to incidence those cases with odds of having not seeked care (1-s)/s | N3=N2+N2*(1-s)/s | DHS 2021 |

Supplementary Table 2: Adjustment of incidence rate for presumed (i.e. untested) cases, reporting rate and health care seeking applied to both DHIS2 and PMI crude incidence.

| **regression coefficient** | **eliminated at step…** | **p-value (F test)** |
| --- | --- | --- |
| IRS:hf_reporting_rate | 1 | 0.969484 |
| IRS:N_hf_active | 2 | 0.940614 |
| N_hf_active | 3 | 0.847019 |
| IRS:tpr_total | 4 | 0.758068 |
| IRS:ipt1_coverage | 5 | 0.616031 |
| IRS:testing | 6 | 0.599217 |
| testing | 7 | 0.360217 |
| hf_reporting_rate | 8 | 0.056271 |
| ipt1_coverage | 9 | 0.180847 |
| IRS | 0 | 8.62E-07 |
| tpr_total | 0 | 0.003838 |

Supplementary Table 3: Model selection of log-linear mixed-effect models of the difference between DHIS2 and PMI incidence using backward step-wise reduction on *model1* (health system covariates only)

| **regression coefficient** | **eliminated at step…** | **p-value (F test)** |
| --- | --- | --- |
| IRS:N_hf_active | 1 | 0.931301 |
| IRS:rainfall | 2 | 0.92298 |
| IRS:biting_rate_hcl_indoor | 3 | 0.884611 |
| Rainfall | 4 | 0.84202 |
| IRS:EIR_ibpn | 5 | 0.80147 |
| IRS:biting_rate_hcl_outdoor | 6 | 0.822069 |
| N_hf_active | 7 | 0.802543 |
| IRS:ipt1_coverage | 8 | 0.642298 |
| IRS:hf_reporting_rate | 9 | 0.632453 |
| IRS:tpr_total | 10 | 6.88E-01 |
| IRS:testing | 11 | 0.66586 |
| Testing | 12 | 0.632313 |
| biting_rate_hcl_indoor | 13 | 0.542917 |
| biting_rate_hcl_outdoor | 14 | 0.521993 |
| IRS:humidity | 15 | 0.404982 |
| IRS:leaf | 16 | 0.648124 |
| Leaf | 17 | 0.501763 |
| Humidity | 18 | 0.32239 |
| IRS:temperature | 19 | 0.211798 |
| Temperature | 20 | 0.392839 |
| IRS | 0 | 9.32E-05 |
| EIR_ibpn | 0 | 0.087861 |
| tpr_total | 0 | 0.000661 |
| hf_reporting_rate | 0 | 0.032704 |
| ipt1_coverage | 0 | 0.047551 |

Supplementary Table 4: Model selection of log-linear mixed-effect models of the difference between DHIS2 and PMI incidence using backward step-wise reduction *model2* (health system covariates as well as environmental variables)

**Supplementary Figure 2:** Bayesian Structural Time Series (BSTS) Causal Framework with Integrated Data Quality Covariates. Adapted from (1).

The state-space model is governed by two core equations:

1. **Observation Equation:** *Yₜ* = *Zₜᵀ αₜ* + *βᵀ Xₜ* + *ϵₜ*, where *ϵₜ* ~ 𝒩(0, *σₑ²*). This connects observed DHIS2 cases (*Yₜ*) to the latent transmission state (*αₜ*) and contemporaneous predictors (*Xₜ*—including reporting completeness, testing rate, test positivity rate, climate drivers, and control district incidence).
2. **State Transition Equation:** *αₜ* = *Tₜ*₋₁ *αₜ*₋₁ + *Rₜ ηₜ*, where *ηₜ* ~ 𝒩(0, *Qₜ*). This models unobserved epidemiological transitions, trends, and seasonality.

The indoor residual spraying (IRS) intervention (*Aₜ*) defines the structural intervention window (*t* ≥ *T*₀). The model learns baseline parameters during the pre-intervention period (*Aₜ* = 0) to project the counterfactual baseline trajectory (*Yₜ*⁽⁰⁾). The pointwise causal impact of IRS is calculated as:

$$\tau_{t}\left( A_{t} \right)=Y_{t}^{\left( 1 \right)}-Y_{t}^{\left( 0 \right)}$$

where *Yₜ*⁽¹⁾ represents the observed post-intervention outcome under IRS (*Aₜ* = 1).

**References**

1.	Brodersen KH, Gallusser F, Koehler J, Remy N, Scott SL. INFERRING CAUSAL IMPACT USING BAYESIAN STRUCTURAL TIME-SERIES MODELS. Ann Appl Stat. 2015;9(1):247–74. Located at: JSTOR

Supplementary Figure 3: Sample run of Bayesian Structural Time Series for Nassian district with PMI data. The panels shows the incidence data (solid lines) vs. BSTS model predictions (dashed lines), before and after the start of the intervention (grey dashed line). Impact is quantified as the average % decrease in incidence after the start of the intervention comparing the recorded data (solid line) to the modeled counterfactual (dashed line). The model in panel A uses only incidence data from Nassian. In panel B, incidence data from both Nassian and the respective control district Dabakala are used. The model in panel C contains incidence data from Nassian, Dabakala and all contemporaneous covariates (see Methods section).
